# Early Diagnostic Prediction of Infective Endocarditis: Development and Validation of EndoPredict-Dx

**DOI:** 10.3390/diagnostics14222547

**Published:** 2024-11-13

**Authors:** Milena Ribeiro Paixão, Bruno Adler Maccagnan Pinheiro Besen, Lucas Zoboli Pocebon, Marilia Francesconi Felicio, Remo Holanda de Mendonça Furtado, Pedro Gabriel Melo de Barros e Silva, Danielle Menosi Gualandro, Marcio Sommer Bittencourt, Tânia Mara Varejão Strabelli, Roney Orismar Sampaio, Flávio Tarasoutchi, Rinaldo Focaccia Siciliano

**Affiliations:** 1 Instituto do Coracao (InCor), Hospital das Clinicas HCFMUSP, Faculdade de Medicina, Universidade de Sao Paulo, Sao Paulo 05508-220, SP, Brazilrinaldo.siciliano@hc.fm.usp.br (R.F.S.); 2 Hospital Israelita Albert Einstein, Sao Paulo 05652-900, SP, Brazil; 3 LIM-51, Departamento de Clínica Médica, Faculdade de Medicina, Universidade de Sao Paulo, Sao Paulo 05508-220, SP, Brazil; 4 Brazilian Clinical Research Institute, Sao Paulo 01404-000, SP, Brazil; 5 Hospital do Coração (HCor), Sao Paulo 59075-050, SP, Brazil; 6 Cardiology Department, Cardiovascular Research Institute Basel (CRIB), University Hospital Basel, 4031 Basel, Switzerland; 7 Department of Internal Medicine and Department of Radiology, University of Pittsburgh Medical Center, Pittsburgh, PA 15219, USA

**Keywords:** infective endocarditis, heart valve disease, decision making, resource-limited settings

## Abstract

Background: Infective endocarditis is a life-threatening disease with diverse clinical presentations, making diagnosis challenging and requiring a range of complementary tests. The level of suspicion, based on clinical judgment, guides decisions regarding the initiation of empirical treatment and the selection of appropriate diagnostic tools. This study aimed to develop and validate the EndoPredict-Dx score for early prediction of infective endocarditis diagnosis. Methods: Patients admitted to a specialized cardiovascular hospital emergency department with suspected infective endocarditis between January 2011 and January 2020 were included. The primary outcome was left-sided infective endocarditis according to the Duke criteria. Logistic regression was used to derive the scoring system, with internal validation performed through bootstrapping. Candidate variables were obtained from the admission medical history, physical examination, and laboratory parameters. Results: Of the 805 individuals with suspected infective endocarditis (median age 56 years (40–73); 58.6% men), 530 confirmed the diagnosis based on the Duke criteria. The EndoPredict-Dx assigned points for male sex, previous endocarditis, petechiae, heart murmur, suspected embolism, symptoms lasting 14 or more days at the time of admission, hemoglobin level ≤ 12 g/dL, leukocyte level ≥ 10 × 10^9^/L, C-reactive protein level ≥ 20 mg/L, and urine red blood cells ≥ 20,000 cells/mL. Patients were divided into three risk groups. The AUROC was 0.78 (95% CI 0.75–0.81) for the derivation cohort and 0.77 for the internal validation. Conclusions: The EndoPredict-Dx score accurately predicted the likelihood of infective endocarditis using clinical and laboratory data collected at admission.

## 1. Introduction

Infective endocarditis is a life-threatening disease with a complex diagnosis due to its significant heterogeneity in clinical presentation, often manifesting with nonspecific symptoms and a reliance on complementary tests for diagnosis [1,2]. According to prior reports, the annual incidence of infective endocarditis is estimated at 1.5 to 15 cases per 100,000 people [2,3,4,5,6,7]. In recent decades, there has been an increasing trend in disease incidence, particularly among older individuals with multiple comorbidities [5,6,7,8]. Despite improvements in infective endocarditis treatment, mortality rates remain high, accompanied by the risk of several complications [9,10,11].

Timely diagnosis and initiation of treatment have the potential to reduce the risk of complications and disease-related mortality in infective endocarditis [12,13]. However, the gold standard for definite diagnosis involves histological analysis of the endocardium, intracardiac tissue, or embolized vegetation obtained postsurgery or during necropsy, and histological analysis is performed in only 20% to 50% of cases [3,4,10]. In this regard, the Duke criteria are the most commonly used diagnostic approach [14,15,16,17]. These include imaging and blood culture results, which may contribute to a time-consuming diagnostic process. Furthermore, studies that evaluated the modified Duke criteria upon admission using echocardiography and blood culture results found a surprisingly low sensitivity [18,19,20]. Although the current trend is to increasingly use advanced imaging techniques for diagnosing endocarditis, such as cardiac computed tomography (CT) and positron emission tomography (PET/CT) [17], these techniques are not always immediately available in emergency settings, particularly in resource-limited settings. Due to these multiple challenges in immediately diagnosing infective endocarditis, a tool to support clinical reasoning could be helpful in the emergency scenario.

Our hypothesis is that a combination of simple and routine data obtained from medical history and usual blood tests, organized into a scoring system, could assist physicians in predicting the likelihood of infective endocarditis within the first hours of admission to the emergency department.

## 2. Materials and Methods

Our target population consisted of patients admitted with suspected infective endocarditis with no other diagnosed infection at the time of clinical suspicion. Data of patients admitted to the emergency department with suspected infective endocarditis between January 2011 and January 2020 were reviewed. The cohort was extracted from a database of consecutively and prospectively selected patients with suspected infective endocarditis, which is part of the hospital infection control unit routine surveillance activities during weekdays.

The inclusion criteria were age 18 years or older and absence of an apparent infectious focus based on the initial clinical and laboratory investigations associated with at least one of the following: (a) a predisposing condition for infective endocarditis (pre-existing valve disease, previous endocarditis, or intravenous drug use) accompanied by one or more of the symptoms documented or reported fever, suspected new embolic event, and New York Heart Association (NYHA) class III or IV acute/subacute heart failure; or (b) fever associated with a murmur and/or suspected systemic embolism in patients without a predisposing condition for infective endocarditis.

Patients with exclusively right-sided endocarditis were excluded due to their distinct predisposing factors and clinical manifestations, which include a lower incidence of systemic embolization and hemodynamic impairment [21,22]. Additionally, patients who had received intravenous antibiotic therapy within 72 h prior to inclusion were excluded, as this could modify laboratory findings, making it more difficult to accurately assess the likelihood of infective endocarditis.

This study was conducted at a specialized tertiary teaching hospital focused on cardiovascular diseases. There are dedicated infectious disease and heart valve disease specialists who collaborate with the emergency department staff. The diagnostic workup for infective endocarditis adhered to the guidelines as they evolved over time [15,16], and cases were reviewed and reclassified according to current practice [17,23].

Data were collected by cardiologists and infectious disease specialists, who evaluated the database from the hospital infection control unit and medical records. Instances with uncertain infective endocarditis diagnoses were scrutinized by a multidisciplinary team. The variables encompassed the following: medical history, physical examination, and laboratory tests during clinical suspicion of infective endocarditis; clinical data during hospitalization; identified microorganisms; and complementary echocardiography and other imaging exams during hospitalization. All heart murmurs were recorded as present or absent regardless of their characteristics. The presence of petechiae was documented if one or more were observed on the conjunctiva or extremities. Suspected embolism was defined by any new neurological deficit (sudden motor, sensory, cognition, speech, or visual impairments) or signs of limb ischemia, which include the absence of a pulse, pallor, cyanosis, pain, or reduced temperature. The variables collected are available in Supplementary Table S1 in the Supplementary Materials.

The primary outcome was the diagnosis of possible or definite left-sided infective endocarditis according to the Duke criteria of the International Society for Cardiovascular Infectious Diseases (ISCVID) published in 2023 [17]. This study was first designed to use modified criteria according to the 2015 guidelines [16], and it was adjusted considering this update. All patients were monitored until hospital discharge or in-hospital death. Patients labeled as “rejected endocarditis” by the Duke criteria also fulfilled the criterion of no readmission within 30 days due to persistent symptoms and/or a subsequent diagnosis of infective endocarditis.

The “Guide for presenting clinical prediction models for use in clinical settings” [24] and “Transparent Reporting of a multivariable prediction model for Individual Prognosis Or Diagnosis” (TRIPOD) statement [25] were used for scoring system construction and reporting (Supplementary Table S2). The included cases and data were entered into the Research Electronic Data Capture (REDCap) electronic system [26].

In line with the recommendations from Riley et al. [27], a sample size of at least 597 patients would be needed to target a margin of error of at most 0.05 around the overall outcome estimate proportion. This was to ensure a small mean absolute error (less than 10%) in the predicted probabilities when the findings were applied in other targeted individuals and to effectively address concerns related to overfitting, considering an outcome proportion of 0.65, 15 parameters, and a Cox–Snell R^2^ of 0.20.

Descriptive statistics stratified by the diagnosis of endocarditis are presented as means and standard deviations or medians and the 25th/75th percentiles for continuous variables, as appropriate for their distribution, and the statistics are presented as counts and percentages for categorical variables. The analysis was performed with *t*-tests or Wilcoxon rank-sum tests for continuous variables, as appropriate, and with the chi-square test for categorical variables.

To create a clinically applicable bedside score, continuous variables of interest were transformed into categorical variables. The cutoff points were chosen based on their nonlinear distribution using restricted cubic splines with 4 knots and based on previously described cutoffs.

Predictor variables were chosen a priori by integrating literature on infective endocarditis predictors [14,15,16,28,29] and expertise from a team composed of cardiologists, infectious disease specialists, and an intensivist. The candidate variables initially selected by the study team were male sex, dialysis status, predisposing condition for infective endocarditis, rheumatic heart disease, previous endocarditis, fever, NYHA functional class III or IV heart failure, petechiae, heart murmur, suspicion of embolism (central nervous system deficit and/or limb ischemia), symptoms initiated 14 or more days prior to admission, hemoglobin level ≤ 12 g/dL, leukocyte count ≥ 10 × 10^9^/L, C-reactive protein (CRP) level ≥ 20 mg/L, red blood cells in the urine ≥ 20,000 cells/mL, and proteinuria ≥ 0.05 g/L.

Variables were incorporated into a preliminary model, and multivariable logistic regression was used with purposeful backward selection of variables for the final model. Then, we constructed a scoring system with points derived by assigning weights to each variable based on the logistic regression beta coefficients. Finally, to assess the diagnostic utility of the model, patients were categorized into three distinct risk levels for developing infective endocarditis by analyzing the numerical and graphical distribution of the risk of this outcome and testing the optimal cutoff point for group differentiation based on the clinical decision needed to be made at the bedside.

We evaluated apparent validation by analyzing the area under the receiver operating characteristic curve (AUROC). Calibration was checked with a calibration belt. The sensitivity, specificity, positive and negative predictive values, and likelihood ratios were calculated for the model and compared with the admission Duke-ISCVID criteria, which utilized information available in the first days following clinical suspicion of endocarditis. We performed bootstrap internal validation with 500 bootstrap resamples for the AUROC, calibration-in-the-large, and calibration slope. 

We compared the performance of the EndoPredict-Dx score with the pre-existing Marseille score for prediction of infective endocarditis diagnosis. As our data collection did not include information on splenomegaly and digital clubbing, we conducted an analysis without scoring these variables.

To address missing data values, we performed multiple imputation with chained equations, including the outcome and the predictors with 10 imputed datasets. For the individuals on dialysis, hematuria and proteinuria were purposefully excluded since the values would not reflect glomerulonephritis, and an indicator variable was assigned to retain the patients in the model.

All tests were 2-sided, and a *p* value < 0.05 was used to indicate statistical significance. No adjustment for multiplicity was performed. Statistical analysis was conducted using Stata SE^®^ software (StataCorp, College Station, TX, USA) version 16.0.

## 3. Results

### 3.1. Descriptive Statistics

In total, 912 suspected cases of infective endocarditis were identified between January 2011 and January 2020. After excluding 107 patients (84 with right-sided infective endocarditis and 29 who had received intravenous antibiotic therapy 72 h before inclusion, with overlap in 6 cases meeting both criteria), the final population for the diagnostic score was comprised of 805 suspected patients with left-sided infective endocarditis. Among them, 530 (65.8%) were diagnosed with infective endocarditis, while 275 (34.2%) were rejected. Among the patients diagnosed with infective endocarditis, 82.3% were classified as definite and 17.7% as possible according to the Duke-ISCVID criteria. No readmissions due to infective endocarditis occurring after discharge were reported within 30 days for participants classified as having rejected infective endocarditis.

The median age of the patients was 56 years (interquartile range, 40 to 73 years), and the majority were men (58.6%). A predisposing risk factor for endocarditis was present in most patients (84.2%), with rheumatic disease being the most common etiology (38.4%), followed by degenerative disease (20.4%) and mitral valve prolapse (8.8%). Fever (67.5%) and murmur (74.8%) were the most common symptoms, with a high proportion of participants (45.6%) presenting with NYHA class III or IV heart failure. The baseline characteristics of the participants are presented in Table 1. We had missing data on creatinine (2 of 805, 0.25%), CRP (3 of 805, 0.57%), hematuria (76 of 805, 9.44%), and proteinuria (77 of 805, 9.57%).

The main clinical characteristics that led to the suspicion of infectious endocarditis were predisposing conditions associated with fever (56.4%), acute NYHA class III or IV heart failure (39.5%), or suspicion of embolism (7.2%). A total of 127 participants (15.8%) were included due to fever associated with murmur and/or suspicion of embolism.

The causative microorganism was identified in 79.6% of infective endocarditis patients. *Streptococcus* spp. from the viridans group constituted the most common agent (26%), followed by *Staphylococcus aureus* (11.5%), *Streptococcus gallolyticus* (8.3%), and *Enterococcus* spp. (7.7%). Upon admission, 81% of patients underwent transesophageal echocardiography, which revealed signs consistent with infective endocarditis in 68% of those classified as possible or definite cases. The findings included vegetations in 55.4% of cases, abscesses in 12.9%, paravalvular regurgitation in 11.9%, valve perforation or rupture in 7.7%, and fistulas in 3.1%. Aortic valve involvement was observed in 54.9% of the patients, while mitral valve involvement was observed in 51.9%.

Emboli suspicion (central nervous system and limbs) occurred in 80 patients with infective endocarditis upon disease suspicion and was confirmed in 63 of them by imaging or pathological specimens from extracted thrombi (78.7%). Emboli were identified in 24.5% of the patients by the end of hospitalization. Therefore, 51.5% of embolic events occurred or presented signs that led to investigation after the first days of admission. The frequency of events was 15.1% in the central nervous system, 8.3% in the spleen, 4.75% in the limbs, 2.5% in the kidneys, 1.5% in the liver, and 0.1% in the vertebrae.

Cardiac surgery was performed in 55.7% of the patients. The overall in-hospital mortality rate of patients diagnosed with infective endocarditis was 30.2%.

### 3.2. Score Derivation and Apparent Validity

The results of the logistic regression analysis, including both univariate and multivariate data, are presented in Supplementary Table S3. The variables and their respective scores are detailed in Table 2.

The probability of infective endocarditis occurrence according to each score and the histogram of the presence/absence of infective endocarditis based on the EndoPredict-Dx score are shown in Supplementary Figures S1 and S2, respectively. Using the likelihood of infective endocarditis for each score point, as depicted in the described graphs, risk groups were established as low risk (0 to 3 points), intermediate risk (4 to 6 points), and high risk of infective endocarditis (≥7 points). The prevalence of infective endocarditis was 16.4%, 46.2%, and 81% for each group, respectively (Table 3).

The EndoPredict-Dx score AUROC was 0.78 (95% CI: 0.75–0.81). The sensitivities of the diagnostic scores for participants at high and intermediate plus high risk were 74.7% and 98.3%, respectively, with specificities of 62.6% and 15.6%, respectively (Table 4). Additionally, the apparent calibration of the diagnostic score demonstrated the use of a well-calibrated scoring system, with excellent agreement between the predicted probabilities and observed outcomes (Supplementary Figure S3).

### 3.3. Internal Validation

Internal validation of the samples was performed using bootstrap resampling with 500 replicates. The bootstrapped C-statistic was 0.77. The bootstrapped calibration-in-the-large, calculated as the difference between the average predicted risk and the overall observed outcome of infective endocarditis, was 0.003 (target value of zero, indicating no systematic bias). The calibration slope assesses the spread of the estimated risk with a target of 1. In our model, the slope was 0.965, indicating that it tends to slightly underestimate the risk of infective endocarditis for higher-risk patients and overestimate the risk for lower-risk patients.

### 3.4. Comparison with Marseille Score and Admission Duke-ISCVID Criteria

**Figure 1:**
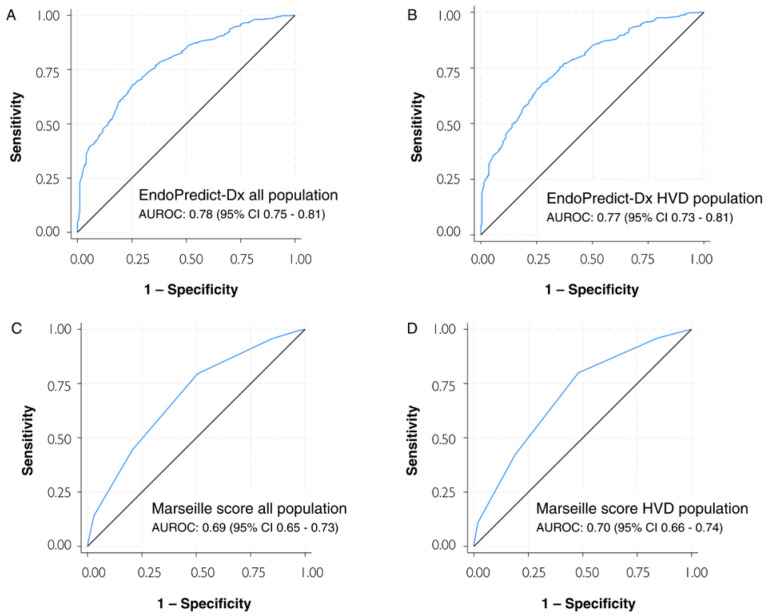
Receiver operating characteristic curve analysis for the prediction of infective endocarditis diagnosis. (**A**) EndoPredict-Dx for the entire study population. (**B**) EndoPredict-Dx for the heart valve disease population. (**C**) Marseille score for the entire population. (**D**) Marseille score for patients with heart valve disease. AUROC, area under the receiver operating characteristic curve; CI, confidence interval; HVD, heart valve disease. The blue line represents the ROC curve and the black line represents the reference line for random classification.

We applied the Marseille score, a pre-existing predictive tool for the diagnosis of infective endocarditis, to assess its performance in both the general population and individuals with valvular disease. Applying the Marseille score to our entire patient database yielded an AUROC of 0.69 (95% CI: 0.65–0.73) and 0.70 (95% CI: 0.66–0.74) for patients with heart valve disease. Figure 1 illustrates the ROC curve comparison of the EndoPredict-Dx and Marseille scores for both populations.

Additionally, we assessed the accuracy of the admission Duke-ISCVID criteria for diagnosing infective endocarditis and compared it with EndoPredict-Dx. As detailed in Table 4, this comparison revealed that the Duke criteria were more specific, whereas the EndoPredict-Dx score demonstrated greater sensitivity.

## 4. Discussion

In our study, the EndoPredict-Dx score was developed to predict the likelihood of infective endocarditis within the first few hours of clinical suspicion in the emergency department. This scoring system only uses clinical features and readily available laboratory tests, making it potentially widely applicable before blood culture and echocardiography results or other imaging modalities are available. The score had good discrimination and calibration based on internal validation. Moreover, the sensitivity of diagnosing infective endocarditis appeared to be higher than that of the Duke-ISCVID criteria, although at the cost of a lower specificity.

Our decisions concerning the variables acknowledge the realities of the emergency department and aim to reflect the real-world diagnostic process, ensuring reproducibility by any physician. Accordingly, we considered all murmurs in our analysis due to the practical challenges in the emergency setting, where distinguishing between new and pre-existing murmurs and accurately classifying murmurs as systolic, diastolic, or “flow” murmurs may not be feasible. Regarding hematuria, our service conducts a quantitative urine analysis, with a cutoff value of 20,000 cells/mL established for our study. In settings where analysis is qualitatively limited, due to the absence of a strict and universal correlation between the number of crosses and the number of red blood cells in the urine, we recommend using the presence of 2+ or more red blood cells in the urine as a strategy for scoring in the EndoPredict-Dx score, ensuring a value above our cutoff is achieved. Following the same logic, echocardiography data were intentionally excluded from the score construction.

Furthermore, we recognize that selecting “suspected embolism” as a variable might raise questions about its reproducibility and accuracy. However, the criteria for suspecting embolism were clearly defined (see data collection methods in Section 2). The decision to use suspected embolism rather than confirmed embolism aligns with our proposal to estimate the individual’s likelihood of having infective endocarditis promptly with minimal resource utilization. Although embolic events may not be confirmed, and emboli could result from intracardiac thrombus, our study confirmed that the presence of suspected central nervous system and limb embolism in patients with suspected infective endocarditis increases the likelihood that the event is related to vegetation embolism. We chose not to include suspected embolism in other locations, such as intra-abdominal organs and the vertebral spine, because symptoms may be more nonspecific and encompass more differential diagnoses.

In our cohort, as a specialized cardiology center, we had many patients at high risk of developing infective endocarditis, including those with prosthetic valves. Although the prevalence of prosthetic valves was high, it did not differ significantly between the endocarditis and rejected cases groups (biological prostheses: 45.7% vs. 49.5%, *p* = 0.31; mechanical prostheses: 6.6% vs. 4.7%, *p* = 0.29). Similarly, the presence of predisposing factors for infective endocarditis did not differentiate between the groups in the multivariate analysis. This suggests that relying solely on these high-risk factors in cases of suspected infective endocarditis is insufficient for distinguishing affected patients. Therefore, incorporating multiple factors, as conducted in the EndoPredict-Dx score, provides a more accurate prediction of the likelihood of infective endocarditis.

In 2008, Richet et al. [28] developed the “Marseille score” to identify individuals with a high probability of infective endocarditis upon hospital admission using data from medical history, physical examination, and laboratory blood tests. Until now, the Marseille score has been the only model developed to predict an infective endocarditis diagnosis based on clinical suspicion. This is a pioneering study with subsequent adjustments and revalidation that exhibits reasonable performance. Our study shares key similarities with the Marseille score, the first being the high prevalence of individuals with pre-existing valvular heart disease: 84.2% in our study versus 59.4% in the original Marseille score [28] and 100% in the 2018 revision of Gouriet et al. [29]. Second, all these models rely solely on clinical and readily available laboratory data. Additionally, a subset of the EndoPredict-Dx score variables, such as male sex, leukocytosis, high CRP level, low hemoglobin, and embolic events, overlapped with the Marseille score. However, there were certain differences. The Marseille score assigned points for finger clubbing and splenomegaly, although finger clubbing was not observed in the validation cohort. In contrast, the EndoPredict-Dx score introduced novel predictors including previous endocarditis, petechiae, heart murmur, symptoms that started 14 or more days prior to admission, and hematuria. The EndoPredict-Dx score presented an AUROC of 0.78, outperforming the Marseille score’s AUROC of 0.703. The validation of the Marseille score in our population exhibited a performance similar to the original study.

The Duke criteria [17] offer limited support for early clinical assessment upon admission to the emergency department. One limitation is the time needed to obtain blood cultures and imaging results, which imposes a significant constraint. Additionally, the criteria may fail to identify cases of infective endocarditis during the first days, demanding additional tests or the occurrence of new clinical events for accurate disease identification. In our cohort, a substantial proportion of embolic events (49.2%) were not suspected or present upon the initial suspicion of infective endocarditis. Further, less than 70% of the echocardiograms performed at admission in patients with infective endocarditis displayed consistent signs of the disease, even though most of the exams (81%) were transesophageal. The absence of signs of infective endocarditis (such as vegetation, abscess, and new prosthesis dehiscence) on the initial echocardiogram maintained the performance of the EndoPredict-Dx score, and the presence of these signs highly increased the chance of the diagnosis, as expected. Hence, we observed a low sensitivity of the Duke criteria in the first days after hospital admission (51.1%), which aligns with the findings of previous studies reporting diagnostic sensitivities ranging from 44% to 52% in the early days after admission [18,19,20]. Therefore, the EndoPredict-Dx score, with its high sensitivity, may serve as an effective initial tool by establishing the level of suspicion for infective endocarditis in early evaluations, while Duke’s criteria offer greater specificity to confirm the diagnosis as additional clinical evidence becomes available. This combined approach could enhance diagnostic accuracy in the early stages, especially in settings where blood culture or imaging results are delayed.

Recently, the Clinical Rule for Infective Endocarditis in the Emergency Department (CREED) score was introduced to predict the likelihood of infective endocarditis among patients with fever in the emergency department [30]. Of the 13,864 patients included, 130 (1%) had endocarditis. The differences between the CREED and EndoPredict-Dx scores stemmed from the differences in their inclusion criteria and study populations. The EndoPredict-Dx score focuses on individuals with specific clinical symptoms indicative of suspected infective endocarditis and more commonly resembles clinical practice. Conversely, the CREED score was designed for a broader and more diverse group of febrile patients admitted to the emergency department with the goal of predicting an endocarditis diagnosis. Moreover, in our case series, 29% of patients did not present with fever, highlighting that the CREED score alone would not be enough for diagnosing infective endocarditis in our population.

The main strengths of our study include a robust sample size and adherence to current guidelines for the development and internal validation of diagnostic and prognostic models. However, since it was conducted at a single specialized tertiary cardiac hospital focused on severe and complex cardiological cases, and involved a younger population compared to those with heart valve disease in high-income countries, the findings may not be fully generalizable to general hospitals due to differences in clinical and microbiological profiles. Additionally, the score is limited to left-sided infective endocarditis, as right-sided cases were purposefully excluded from the study due to their distinct predisposing factors and clinical manifestations [21,22]. Therefore, external validation in diverse populations is required before the EndoPredict-Dx score can be widely applied in broader clinical settings.

In summary, the EndoPredict-Dx score predicted the diagnosis of infective endocarditis early among patients admitted to the emergency department. This scoring system has the potential to aid in decision making that relies exclusively on clinical and widely available laboratory tests.

## Figures and Tables

**Table 1:** Clinical characteristics of the patients at baseline.

Variables	Total (*n* = 805)	Rejected Endocarditis (*n* = 275)	Endocarditis (*n* = 530)	*p* Value
Demography				
Median age—years (IQR *)	56 (40–73)	57 (41–73)	56 (39–73)	0.49
Male sex—*n* (%)	472 (58.6)	135 (49.1)	337 (63.6)	<0.001
Comorbidities—*n* (%)				
Hypertension	424 (52.7)	146 (53.1)	278 (52.5)	0.86
Diabetes	148 (18.4)	49 (17.8)	99 (18.7)	0.76
Nondialytic chronic kidney disease (CrCl ^†^ < 60 mL/min)	79 (9.8)	25 (9.1)	54 (10.2)	0.62
Dialysis	43 (5.3)	9 (3.3)	34 (6.4)	0.060
Pre-existing heart valve disease				
Rheumatic	309 (38.4)	123 (44.7)	186 (35.1)	0.008
Degenerative	164 (20.4)	55 (20)	109 (20.6)	0.85
Mitral prolapse	71 (8.8)	16 (5.8)	55 (10.4)	0.031
Bicuspid aortic valve	35 (4.3)	6 (2.2)	29 (5.5)	0.030
Noncyanogenic congenital heart disease	17 (2.1)	6 (2.2)	11 (2.1)	0.92
Cyanogenic congenital heart disease	16 (2)	5 (1.8)	11 (2.1)	0.80
Biological prosthesis	378 (47)	136 (49.5)	242 (45.7)	0.31
Mechanical prosthesis	48 (6)	13 (4.7)	35 (6.6)	0.29
Ascending aorta prosthetic graft or other nonvalvular intracardiac prosthesis	9 (1.1)	0 (0)	9 (1.7)	0.030
TAVI ^‡^	1 (0.1)	0 (0)	1 (0.2)	0.47
Unknown cause	67 (8.3)	27 (9.8)	40 (7.5)	0.27
Intravenous drug	3 (0.4)	0 (0)	3 (0.6)	0.21
Previous endocarditis	136 (16.9)	39 (14.2)	97 (18.3)	0.14
Intracardiac devices	60 (7.5)	26 (9.5)	34 (6.4)	0.12
Dialysis catheter	30 (3.7)	8 (2.9)	22 (4.2)	0.38
Predisposing risk factor for endocarditis ^§^	678 (84.2)	243 (88.4)	435 (82.1)	0.037
Signs and symptoms—*n* (%)				
Fever	543 (67.5)	165 (60.0)	378 (71.3)	0.001
Acute NYHA ^||^ class III or IV heart failure	367 (45.6)	150 (54.5)	217 (40.9)	<0.001
Petechiae	54 (6.7)	5 (1.8)	49 (9.2)	<0.001
Heart murmur	602 (74.8)	169 (61.5)	433 (81.7)	<0.001
Suspected emboli (CNS ^#^ deficit or limb ischemia signs)	92 (11.4)	4 (1.5)	130 (24.5)	<0.001
Symptoms lasting 14 or more days prior to admission	378 (47)	100 (36.4)	278 (52.5)	<0.001
Laboratory tests				
Hemoglobin ≤ 12 g/dL—*n* (%)	492 (61.1)	125 (45.5)	367 (69.2)	<0.001
Leukocyte ≥ 10 × 10^9^/L—*n* (%)	408 (50.7)	116 (42.2)	292 (55.1)	<0.001
Platelets–×10^9^/L (IQR *)	198 (139–278)	198 (139–280)	197 (139–273)	0.51
C-reactive protein ≥ 20 mg/L—*n* (%) **	702 (87.5)	213 (77.5)	498 (92.8)	<0.001
Creatinine–mg/dL—*n* (%) ^††^	1.17 (0.92–1.60)	1.19 (0.93–1.61)	1.16 (0.91–1.59)	0.49
Urine tests—*n* (%)				
Protein ≥ 0.05 g/L ^‡‡^	235 (34.3)	72 (29.4)	163 (37.0)	0.043
Red blood cells ≥ 20,000 cells/mL ^§§^	165 (24.1)	37 (15.2)	128 (29.0)	<0.001

* IQR, interquartile range; ^†^ CrCl, creatinine clearance; ^‡^ TAVI, transcatheter aortic valve implantation; ^§^ predisposing risk factor for endocarditis: pre-existing left heart valve disease, previous endocarditis, or intravenous drug use; ^||^ NYHA, New York Heart Association; ^#^ CNS, central nervous system; ** *n* = 802; ^††^ *n* = 803; ^‡‡^ *n* = 728; ^§§^ *n* = 729.

**Table 2:** EndoPredict-Dx score for the prediction of left-sided infective endocarditis diagnosis.

Variable	Scores
Male sex	+1
Previous infective endocarditis	+1
Petechiae	+2
Heart murmur	+2
Suspected emboli (CNS * deficit or limb ischemia signs)	+5
Symptoms lasting 14 or more days at the time of admission	+1
Hemoglobin ≤ 12 g/dL	+2
Leukocyte ≥ 10 × 10^9^/L	+1
C-reactive protein ≥ 20 mg/L	+2
Red blood cells, urine ≥ 20,000 cells/mL	+1

* CNS, central nervous system.

**Table 3:** Risk stratification of left-sided infective endocarditis diagnosis according to the EndoPredict-Dx score.

Risk Group	Scores	Number of Participants—*n* (%)	Number of Infective Endocarditis—*n* (%)
Low	0 to 3	55 (6.8)	9 (16.4)
Intermediate	4 to 6	249 (30.9)	115 (46.2)
High	≥7	501 (62.2)	406 (81)
Total	0 to 18	805 (100)	530 (65.8)

**Table 4:** Accuracy of diagnosis for left-sided infective endocarditis using Duke criteria upon admission versus the high and intermediate–high probability categories of the EndoPredict-Dx score.

Score	Sensitivity% (95% CI *)	Specificity% (95% CI *)	NPV% ^†^ (95% CI *)	PPV% ^‡^ (95% CI *)	LR+ ^§^	LR- ^‖^
Admission Duke	51.1 (47.5–54.6)	99.3 (98.4–99.7)	99.3 (98.4–99.7)	51.3 (47.8–54.8)	70.3	0.49
EndoPredict-Dx: high risk	74.7 (71.5–77.6)	62.6 (59.2–66.0)	56.2 (52.6–59.6)	79.4 (76.4–82.1)	1.99	0.40
EndoPredict-Dx: Intermediate–high risk	98.3 (97.1–99.0)	15.6 (13.2–18.3)	82.7 (79.9–85.3)	69.2 (65.9–72.4)	1.17	0.11

* CI, confidence interval; ^†^ NPV, negative predictive value; ^‡^ PPV, positive predictive value; ^§^ LR+: positive likelihood ratio; ^‖^ LR-: negative likelihood ratio.

## Data Availability

The partial or complete de-identified dataset, along with the data dictionary and analytic code for this investigation, can be provided to researchers seeking collaboration upon appropriate ethical approval. Interested parties can contact the corresponding author.

## Supplementary Materials

The following supporting information can be downloaded at https://www.mdpi.com/article/10.3390/diagnostics14222547/s1, Table S1: Data collection details; Table S2: TRIPOD Checklist: Prediction Model Development; Table S3: Factors associated with the diagnosis of left-sided infective endocarditis: univariate and multivariable logistic regression of the EndoPredict-Dx score; Figure S1: Likelihood of left-sided infective endocarditis occurrence according to the EndoPredict-Dx score; Figure S2: Histogram of the EndoPredict-Dx score in confirmed and rejected endocarditis patients; Figure S3: Calibration of the EndoPredict-Dx Score for Predicting Left-Sided Infective Endocarditis Diagnosis.
